# Heterologous Expression of Three *Ammopiptanthus mongolicus* Dehydrin Genes Confers Abiotic Stress Tolerance in *Arabidopsis thaliana*

**DOI:** 10.3390/plants9020193

**Published:** 2020-02-05

**Authors:** Hongwei Cui, Yang Wang, Tingqiao Yu, Shaoliang Chen, Yuzhen Chen, Cunfu Lu

**Affiliations:** 1 Beijing Advanced Innovation Center for Tree Breeding by Molecular Design, National Engineering Laboratory for Tree Breeding, College of Biological Sciences and Biotechnology, Beijing Forestry University, Beijing 100083, China; 2 College of Life Science, Pecking University, Beijing 100083, China

**Keywords:** *Ammopiptanthus mongolicus*, dehydrin, abiotic stress tolerance, transgenic plants

## Abstract

*Ammopiptanthus mongolicus*, a xerophyte plant that belongs to the family Leguminosae, adapts to extremely arid, hot, and cold environments, making it an excellent woody plant to study the molecular mechanisms underlying abiotic stress tolerance. Three dehydrin genes, *AmDHN132*, *AmDHN154*, and *AmDHN200* were cloned from abiotic stress treated *A. mongolicus* seedlings. Cytomembrane-located AmDHN200, nucleus-located AmDHN154, and cytoplasm and nucleus-located AmDHN132 were characterized by constitutive overexpression of their genes in *Arabidopsis thaliana*. Overexpression of AmDHN132, AmDHN154, and AmDHN200 in transgenic Arabidopsis improved salt, osmotic, and cold tolerances, with AmDHN132 having the largest effect, whereas the growth of transformed plants is not negatively affected. These results indicate that AmDHNs contribute to the abiotic stress tolerance of *A. mongolicus* and that *AmDHN* genes function differently in response to abiotic stresses. Furthermore, they have the potential to be used in the genetic engineering of stress tolerance in higher plants.

## 1. Introduction

Dehydrins (DHNs), also known as group 2 late embryogenesis abundant (LEA) proteins [1,2,3,4,5,6], accumulate in response to dehydration stresses and play protective roles under stress conditions [7,8,9]. They function to resist drought, low temperature, high salinity, and abscisic acid stress. Dehydrins are typically highly hydrophilic and possess a large fraction of polar and charged amino acids [10]. The DHN protein family is characterized by the existence of a K-segment [11], while other conserved motifs, such as S-, Y-, and Φ-segments, have also been found in this protein family. However, the number of common amino acid sequences and the specific motifs in these proteins differ [12,13]. All plant dehydrins contain 1–15 K-segments, whose sequence is EKKGIMDKIKEKLPG. K-segments are lysine-rich and are located near the C terminus of dehydrins [14,15]. K-segments in dehydrins play an essential role, mainly stabilizing membranes [16,17,18]. Another conserved motif near the N-terminus of dehydrins is the tyrosine-rich Y-segment, which consists of 1–35 tandem repeats of the consensus sequence, (V/T)D(E/Q)YGNP. Another relatively conserved dehydrin sequence is the S-segment consisting of 4–10 serine residues [11]. The number and sequence of the K-, Y-, and S-segments define diverse subclasses: Kn, SKn, KnS, YnKn, and YnSKn [19].

Accumulating evidence shows a positive correlation between dehydrin proteins and abiotic stress tolerance. Overexpression of the *Opuntia streptacantha OpsDHN1* gene in *Arabidopsis thaliana* increased its tolerance to freezing treatment [20]. The dehydrin gene, *OesDH*, isolated from oleaster, the wild form of olive, enhanced drought tolerance in Arabidopsis transgenic plants [21]. Moreover, overexpression of durum wheat *DHN-5* in Arabidopsis confers salinity tolerance [22]. Overexpression of *RcDhn5* from *Rhododendron catawbiense* in Arabidopsis plants improved its freezing tolerance [23].

Although dehydrins participate in stress tolerance in cyanobacteria [24], *Physcomitrella patens* [25], and the necrotrophic fungus *Alternaria brassicicola* [26], the functions of DHN orthologs are poorly characterized in non-model plants, particularly perennial woody plants. *Ammopiptanthus mongolicus*, an evergreen broadleaf shrub plant [27,28], plays important ecological roles in the Inner Mongolia desert of China, and is an ideal species for research into abiotic tolerance of trees. We previously cloned and assessed the function of a cold-induced dehydrin-like gene, *AmCIP*, from *A. mongolicus* [6,29]. Here, we cloned *AmDHN132*, *AmDHN154*, and *AmDHN200* from *A. mongolicus* via their high homology with *AmCIP*. Overexpression of the three dehydrin genes increased the tolerance of transgenic Arabidopsis to salt, osmotic, and cold stresses.

## 2. Results

### 2.1. Sequence Characterization of the Dehydrin Gene Family

**Figure 1:**
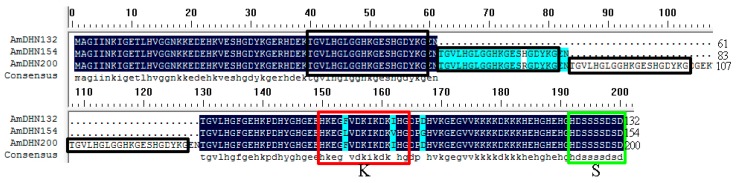
Multiple sequence alignment of AmDHNs. Features include one K-segment (red box) and one S-segment (green box). The repeated fragments are displayed in black boxes.

On the basis of previous studies [6,29], PCR amplification and sequencing indicated that three dehydrin cDNAs were successfully cloned and that each had a full-length open reading frame. These clones were 399, 465, and 603 bp in length, encoding 132, 154, 200 amino acids, respectively (Figure 1, Table 1) and were named AmDHN132, AmDHN154, and AmDHN200. The cDNAs were uploaded to GenBank with the login numbers MH512938 for AmDHN132, MH512939 for AmDHN154, and MH512941 for AmDHN200.

The AmDHNs were classified as SK-types because of the following structural characteristics. They contain one S-segment and one K-segment and no Y-segment. However, one segment containing 20 amino acid residues, TGVLHGLGGHKGES(H/R)GDYKG, repeats one, two, and four times in AmDHN132, AmDHN154, and AmDHN200, respectively.

### 2.2. Creation of Transgenic Arabidopsis and the Subcellular Localization of AmDHNs

**Figure 2:**
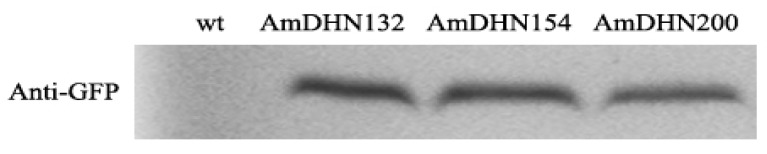
Expression of AmDHN132, AmDHN154, and AmDHN200 in transgenic Arabidopsis detected by Western blot analysis.

**Figure 3:**
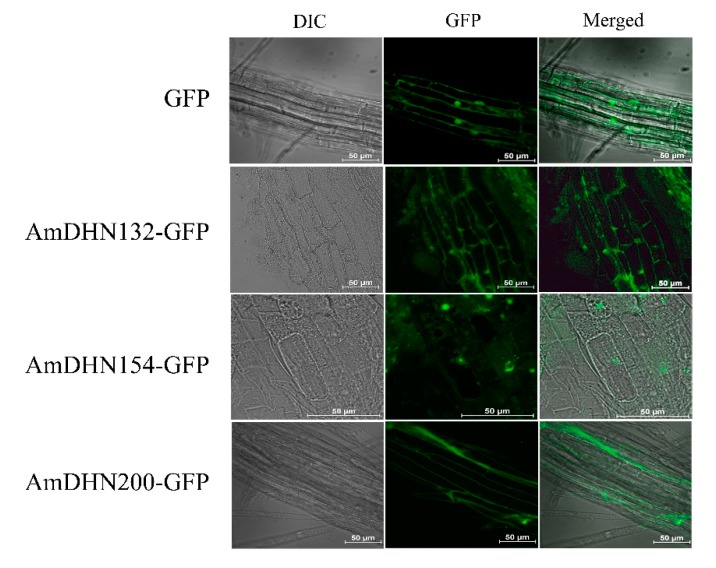
Subcellular localization of AmDHN132-GFP, AmDHN154-GFP and AmDHN200-GFP fusion proteins in transgenic Arabidopsis root cells. Bar = 50 μm.

AmDHNs overexpression vectors (Figure S4) were constructed and transformed into Arabidopsis. Western blotting indicated that the three AmDHN genes were successfully expressed in transgenic Arabidopsis (Figure 2). GFP fluorescence showed that AmDHN154 localized in the nucleus, AmDHN200 localized in the cell membrane, and AmDHN132 localized in the cytoplasm and nucleus (Figure 3).

### 2.3. Heterologous Expression of AmDHNs in Arabidopsis Enhances Abiotic Stress Tolerance

**Figure 4:**
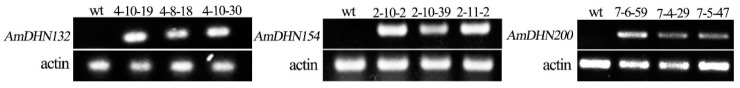
RT–PCR analysis of the expression of *AmDHNs* in transgenic Arabidopsis plantlets. The transgenic lines are indicated at the top of each lane. wt represents wild-type plants. 4-8-18, 4-10-19, and 4-10-30 represent *AmDHN132* transgenic lines. 2-10-2, 2-10-39, 2-11-2 represent *AmDHN154* transgenic lines. 7-6-59, 7-4-29, and 7-5-47 represent *AmDHN200* transgenic lines.

RT-PCR showed that *AmDHNs* were expressed in transgenic plants (Figure 4). To demonstrate whether *AmDHNs* are involved in tolerance to abiotic stresses, wild-type and transgenic Arabidopsis overexpressing *AmDHN132*, *AmDHN154*, or *AmDHN200* were treated with NaCl, mannitol, or low temperature.

**Figure 5:**
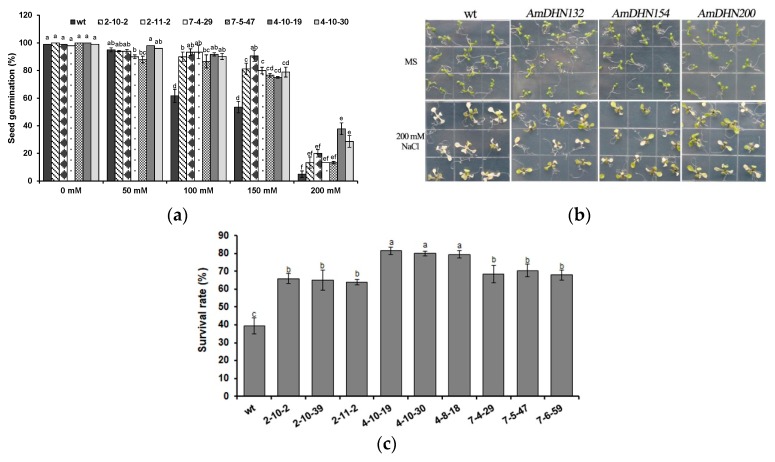
Ectopic expression of *AmDHNs* in Arabidopsis affects salt tolerance; (**a**) seed germination rates of the wild type and *AmDHN* transgenic plants under different concentrations of NaCl; (**b**) growth performance of the wild type and *AmDHN* transformants grown under normal and salt treatment conditions; (**c**) survival rate of salt-treated plants. *AmDHN154* transformed lines: 2-10-2, 2-10-39, 2-11-2. *AmDHN132* transformed lines: 4-10-19, 4-10-30, 4-8-18. *AmDHN200* transformed lines: 7-4-29, 7-5-47, 7-6-59. Each column is the mean of three independent experiments and bars represent the standard error of the mean. The different letters denote significant differences (*p* < 0.05) according to ANOVA results.

Wild-type and transgenic seeds were germinated on MS medium containing 0 (control), 50, 100, 150, or 200 mM NaCl. Under normal conditions, there was no difference between the transgenic and wild-type plants, but the seed germination rate of transgenic plants was significantly higher than that of wild-type plants on NaCl-containing medium (Figure 5a). After 10 days of treatment with 200 mM NaCl, most of the wild-type Arabidopsis leaves became white and gradually died, but overall the transgenic Arabidopsis grew better than wild-type Arabidopsis (Figure 5b). The seedling survival rate of the transgenic Arabidopsis was higher than that of the wild type (Figure 5c). The data also indicated that *AmDHN132* was better than *AmDHN154* and *AmDHN200* in conferring salt resistance.

**Figure 6:**
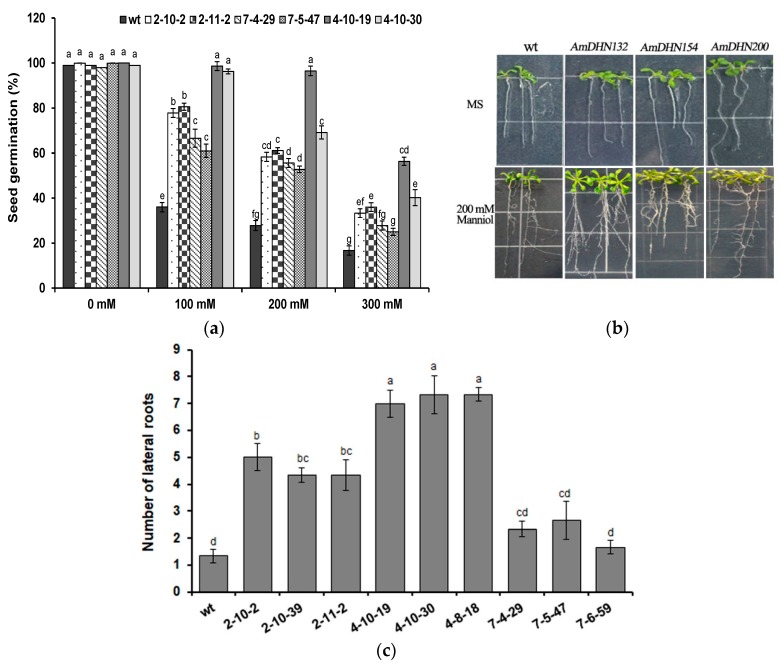
Overexpression of AmDHNs in Arabidopsis affects tolerance to osmotic stress. (**a**) Seed germination rates of wild-type and AmDHN transgenic plants under different concentrations of mannitol. (**b**) Phenotypes of the wild-type (wt) and AmDHN154, AmDHN132, and AmDHN200 overexpressing lines with and without osmotic stress. (**c**) Lateral root numbers of wild-type and transgenic plants after osmotic treatments. AmDHN154 transformed lines: 2-10-2, 2-10-39, and 2-11-2. AmDHN132 transformed lines: 4-10-19, 4-10-30, and 4-8-18. AmDHN200 transformed lines: 7-4-29, 7-5-47, and 7-6-59. Each column is the mean of three independent experiments and bars represent the standard error of the mean. The different letters denote significant differences (*p* < 0.05) according to ANOVA results.

To reveal the role of *AmDHNs* in resistance to osmotic stress, mannitol (0–300 mM) was added to MS medium and seed germination was detected. *AmDHNs* improved seed germination under osmotic stress (Figure 6a). After 10 days of treatment with 200 mM mannitol, the number of lateral roots of transgenic Arabidopsis was much higher than that of the wild type. When grown on normal medium, the roots of *AmDHN200* transgenic Arabidopsis were well developed and the true leaves were larger than those of the wild type (Figure 6b). The number of lateral roots of transgenic Arabidopsis was higher than that of the wild type after osmotic stress (Figure 6c). Overall, *AmDHN132* was more effective than *AmDHN154* and *AmDHN200* in improving osmotic resistance.

**Figure 7:**
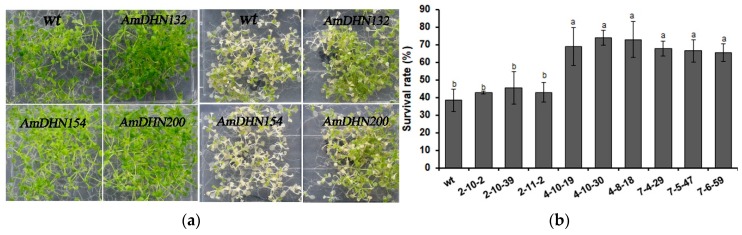

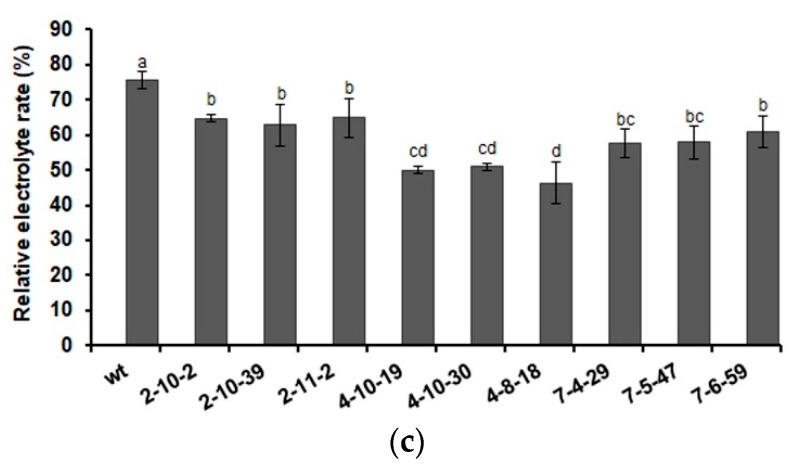
Freezing tolerance of wild-type and transgenic Arabidopsis plantlets. (**a**) Phenotype of wild-type and *AmDHN154*, *AmDHN132*, and *AmDHN200* transgenic plants under normal conditions (left); and their responses to freezing stress (right). (**b**) Survival rate after recovery from freezing (−5 °C). (**c**) Electrolyte leakage rate after recovery from freezing (−5 °C). *AmDHN154* transformed lines: 2-10-2, 2-10-39, and 2-11-2. *AmDHN132* transformed lines: 4-10-19, 4-10-30, and 4-8-18. *AmDHN200* transformed lines: 7-4-29, 7-5-47, and 7-6-59. Each column is the mean of three independent experiments and bars represent the standard error of the mean. The different letters denote significant differences (*p* < 0.05) according to ANOVA results.

Freezing tolerance was also evaluated. After treatment at −5 °C followed by recovery, the growth phenotype, electrolyte leakage, and survival rate showed that overexpression of *AmDHN132* and *AmDHN200* significantly improved the freezing resistance of transgenic Arabidopsis (Figure 7).

## 3. Discussion

### 3.1. AmDHNs Are Conserved in A. mongolicus 

Dehydrins are classified as group II LEA family proteins and they play a fundamental role in resisting abiotic and biotic stresses [30]. The DHNs is a redundant family with 4 to 13 or more genes in different plant species [31]. The sequence of dehydrin proteins must have at least one copy of the lysine-rich K-segment [9]. Accordingly, in this study, we amplified three dehydrin genes from *A. mongolicus* based on previous findings [29]. AmDHN members are highly homologous and contain one S-segment and one K-segment. Unlike most dehydrins, the K- and S-segments are only present in single copies in the AmDHN sequences and the S-segment is located at the C-terminus, making them KS-type dehydrins [32]. In addition, the AmDHN sequences differ in the number of repeats of a 20 amino acid residue sequence, TGVLHGLGGHKGES(H/R)GDYKG.

### 3.2. Overexpression of AmDHNs Increases Abiotic Stress Tolerance of Arabidopsis

Research indicates that the role of DHNs is to defend the cell from dehydration damage caused by environmental stresses and cellular dehydration [33]. Stress tolerance was improved in tobacco by overexpression of SbDhn1 from *Sorghum bicolor* [34], in bananas by constitutive overexpression of MusaDHN-1 [35], and in rice by overexpression of OsDhn1 [36]. Here, we found that constitutive expression of *AmDHN* genes from *A. mongolicus* in Arabidopsis increased salt, osmotic, and cold tolerance, which is consistent with these previous studies. However, functional differences exist among the AmDHNs. Previously, Falavigna et al. [37] found that apple MdDHN proteins have different functions and overlapping levels of expression, and that their expression is fine-tuned by the environment during the dormancy process. In this study, Arabidopsis seedlings overexpressing three AmDHN genes have different abiotic stress tolerances, which may result from their differences in structure, homology, and location.

DHNs are localized in different cell compartments, such as the cytosol, nucleus, mitochondria, vacuoles, and close to the plasma membrane, but they are mainly localized in the cytoplasm and nucleus [38]. In some cases, dehydrin proteins are limited to certain areas of an organ, such as guard cells, meristem cells, and pollen sacs [38]. Correspondingly, differences in dehydrin localization may be one of the reasons for its functional differentiation. AmDHN132 and AmDHN200 proteins in the transgenic Arabidopsis were located in the cell membrane (Figure 2). The plants transformed by these genes showed high survival rates and lower conductivity values after freezing (Figure 6). The degree of extravasation of electrolyte reflects the relative integrity of the cell membrane, indicating that AmDHN132 and AmDHN200 protected cell membranes from freezing injury. Some studies reported that DHN accumulates or migrates to the plasma membrane during stress [39,40]. The membrane damage of *DHNA* and *DHNB* knockout mutants of *P. patens* increased under stress [41]. Our findings support the membrane-protective effect of dehydrin proteins.

Although we have no information on the upstream cis-acting elements of the three *AmDHN* genes, they will be important in the functional differentiation of the three genes. Agarwal et al. [42] found that *PpDHNA* and *PpDHNB* were resistant to drought and cold, and they identified upstream low-temperature response cis-acting elements in the genes. By contrast, *PpDHNC* showed microbial resistance, and its upstream cis-acting elements were related to biological stress and not low-temperature. The difference among the upstream sequences of these three genes therefore led to functional differentiation. The identification of upstream cis-acting regulatory elements of the *AmDHN* genes is therefore important and will help reveal the molecular mechanism of AmDHNs underlying abiotic stress in *A. mongolicus*.

### 3.3. Potential Use of AmDHNs and Further Research 

In summary, overexpression of *AmDHNs* improves the abiotic tolerance in the transgenic Arbidopsis plants, where the growth of transformed plants is not negatively affected. We, therefore, believe that *AmDHNs* could potentially be used in diverse crop and tree species to confer abiotic stress tolerance. For further research into the biological functions of AmDHNs, visualization of their changing localization under stress conditions will be highly valuable, because the differences in dehydrin localization and migration may be one of the reasons for its functional differentiation.

## 4. Materials and Methods 

### 4.1. Plant Material and Normal and Stress Growth Conditions

*A. mongolicus* seeds were sown in pots with potting perlite matrix and grown in a culture chamber (25 °C, 60% relative humidity, 16 h light/8 h dark). Two-week-old seedlings were selected for experiments.

For cold treatment, two-week-old *A. mongolicus* seedlings were developed at 4 °C for 1, 2, 4, 8, and 16 days. For drought treatment, seedlings were transferred to 20 × 20 cm Petri dishes and incubated at 25 °C in 60% humidity for 1, 2, 3, and 4 days. For salt treatment, pots were irrigated with 200 mL 1% NaCl solution and incubated at 25 °C, in 70% humidity with 16 h light/8 h dark for 1, 2, 4, 8, and 16 days. For heat treatment, seedlings were developed at 40 °C for 1, 2, 4, 8, 12, and 24 h. All harvested seedlings were stored at −80 °C until use.

### 4.2. Cloning A. mongolicus Dehydrin Genes

Primers PCAN (5′-CGGGGTACCATGGCAGGTATCATCAACAAGATTG-3′) and PCBP (5′-CGCGGATCCCTAGTCACTGTCACTGCTGCTGCTG-3′) were designed according to the *AmCIP* sequence (Figure S1) [29]. Totally, 126 cDNAs were cloned by PCR from cold, drought, salt, and high temperature treated *A. mongolicus* seedlings (Figure S2). After sequencing and eliminating repetitive sequences, we used CD-HIT online software under default parameters (sequence identity cut-off = 0.9, word length = 5) to cluster similar proteins into clusters that met a user-defined similarity threshold. We selected three representative members and named them *AmDHN132, AmDHN154,* and *AmDHN200* (Figure S3).

### 4.3. Construction and Transformation of Eukaryotic Expression Vectors

Using gene specific primers, PCAN and PCBP, the open reading frames of *AmDHNs* were amplified by PCR. To construct the expression vectors, the pEASY^®^-Blunt Simple Cloning vector containing the ORF of *AmDHNs* was used as a template and a Hind III restriction site was added to the 5’end of the sense primers, a Pst I site was added to the 5’end of the antisense primer. The PCR product was digested with Hind III/Pst I and was subcloned into pCAMBIA2300-GFP vector, which uses the cauliflower mosaic virus 35S promoter to drive expression (Figure S4). Recombinant and empty plasmids were transferred to the *Agrobacterium* strain LBA4404 and used to transform *A. thaliana* by the floral dip method [43].

### 4.4. Screening of Homozygous Lines

T1 seeds were collected, surface sterilized, and then seeded on sterile media containing 50 μg/mL kanamycin to select positive seedlings. *A. thaliana* was grown in a culture chamber after a 2-day vernalization period. Plants were then transferred to soil for cultivation to obtain T2 seeds. T3 seeds were acquired in the same way. Finally, T3 generation seeds grown on medium containing kana were considered to be homozygous strains if they grew normally.

### 4.5. RT-PCR Analysis

RT-PCR was performed according to the manufacturer’s instructions (Tiangen, Biotech Co. LTD, Beijing, China). Sample and control reactions were amplified for 30 cycles. Primers used are listed in Table S1. Actin was used as an internal control in semi-quantitative RT-PCR analysis of dehydrin genes.

### 4.6. Western Blot Analysis

AmDHN protein levels were detected by Western blotting [44]. Proteins were extracted from two-week-old leaves, separated by 12% SDS-PAGE, and then transferred to nitrocellulose membranes (Amersham Biosciences, Piscataway, NJ, USA). Blots were probed using an anti-GFP tag monoclonal antibody HRP-goat anti-mouse IgG (Augct, Beijing, China). Signals were detected by chemiluminescence using the chemiluminescent horseradish peroxidase (HRP) substrate system (Millipore) [7].

### 4.7. Subcellular Localization of AmDHN

Arabidopsis seeds were vernalized at 4 °C for 2 days and then grown on MS solid medium in the culture room for 4 days. To localize AmDHNs, fluorescence of GFP in the root cells was observed by laser confocal microscopy (Ti-E/Ti-U/Ti-S, Nikon, Tokyo, Japan). For GFP, the excitation wavelength was 488 nm.

### 4.8. Abiotic Stress Tolerance Analysis

Sterilized control and transgenic Arabidopsis seeds were vernalized at 4 °C on square MS (pH 5.8) medium for 2 days and then grown in a culture chamber under long-day conditions at 22 °C. For freezing tolerance analysis, 10-day-old wild-type and homozygous transgenic Arabidopsis plants grown under normal culture conditions were transferred to −5 °C for 4 h and then survival rate was calculated after 3 days of recovery at 22 °C. For electrolyte leakage analysis, eight pieces of leaf samples with the same area from each group were washed with deionized water and then immersed in 10 mL of deionized water. After vacuum filtration, the electrical conductivity of the supernatant (S1) was detected using a DDS-307 detector. The samples were then lethally boiled to detect the ultimate conductivity (S2, maximum conductivity of tissues). The relative leakage degree was calculated by the ratio S1/S2. For salt and drought stress tolerance evaluation, wild-type and transgenic Arabidopsis seeds were cultured on MS medium containing 0 (control), 50, 100, 150, 200 mM NaCl or 100, 200, 300 mM mannitol for 10 days. Germination rates were then measured. Eight-day-old seedlings were moved to MS medium containing 200 mM NaCl or 200 mM mannitol and cultured for 10 days. The survival rate was calculated after stress treatment. The experiment was repeated three times.

### 4.9. Data Analysis

Experiments were conducted in three biological replicates with each replicate consisting of three plants. All data were analyzed using SPSS (version 19.0; IBM Corp., Armonk, NY, USA) and were expressed as the mean ± SE. Multigroup comparisons of the means were carried out by one-way analysis of variance (ANOVA) test with post hoc contrasts by Student–Newman–Keuls test. The statistical significance for all tests was set at *p* < 0.05. All charts were plotted using Microsoft Excel.

## Figures and Tables

**Table 1:** Characteristics of the three dehydrin genes.

Gene Name	Gene Size (bp)	Protein Length (aa)	Molecular Weight (kD)	Gene Accession Number
*AmDHN132*	399	132	14.41	MH512938
*AmDHN154*	465	154	16.65	MH512939
*AmDHN200*	603	200	21.42	MH512941

## Supplementary Materials

The following are available online at https://www.mdpi.com/2223-7747/9/2/193/s1, Figure S1: cDNA sequence of *AmCIP*, Figure S2: *AmDHN*s (cDNA) were cloned by PCR from cold, drought, salt, and heat treated *A. mongolicus* seedlings, Figure S3a: cDNA sequence of *AmDHN132*, Figure S3b: cDNA sequence of *AmDHN154*, Figure S3c: cDNA sequence of *AmDHN200*, Table S1: List of primers used, Figure S4: AmDHNs overexpression vectors.
